# Head Movements Evoked in Alert Rhesus Monkey by Vestibular Prosthesis Stimulation: Implications for Postural and Gaze Stabilization

**DOI:** 10.1371/journal.pone.0078767

**Published:** 2013-10-17

**Authors:** Diana E. Mitchell, Chenkai Dai, Mehdi A. Rahman, Joong Ho Ahn, Charles C. Della Santina, Kathleen E. Cullen

**Affiliations:** 1 Department of Physiology McGill University, Montreal, Quebec, Canada; 2 Department of Otolaryngology, Head & Neck Surgery Johns Hopkins University School of Medicine, Baltimore, Maryland, United States of America; Tokai University, Japan

## Abstract

The vestibular system detects motion of the head in space and in turn generates reflexes that are vital for our daily activities. The eye movements produced by the vestibulo-ocular reflex (VOR) play an essential role in stabilizing the visual axis (gaze), while vestibulo-spinal reflexes ensure the maintenance of head and body posture. The neuronal pathways from the vestibular periphery to the cervical spinal cord potentially serve a dual role, since they function to stabilize the head relative to inertial space and could thus contribute to gaze (eye-in-head + head-in-space) and posture stabilization. To date, however, the functional significance of vestibular-neck pathways in alert primates remains a matter of debate. Here we used a vestibular prosthesis to 1) quantify vestibularly-driven head movements in primates, and 2) assess whether these evoked head movements make a significant contribution to gaze as well as postural stabilization. We stimulated electrodes implanted in the horizontal semicircular canal of alert rhesus monkeys, and measured the head and eye movements evoked during a 100ms time period for which the contribution of longer latency voluntary inputs to the neck would be minimal. Our results show that prosthetic stimulation evoked significant head movements with latencies consistent with known vestibulo-spinal pathways. Furthermore, while the evoked head movements were substantially smaller than the coincidently evoked eye movements, they made a significant contribution to gaze stabilization, complementing the VOR to ensure that the appropriate gaze response is achieved. We speculate that analogous compensatory head movements will be evoked when implanted prosthetic devices are transitioned to human patients.

## Introduction

**Figure 1:**
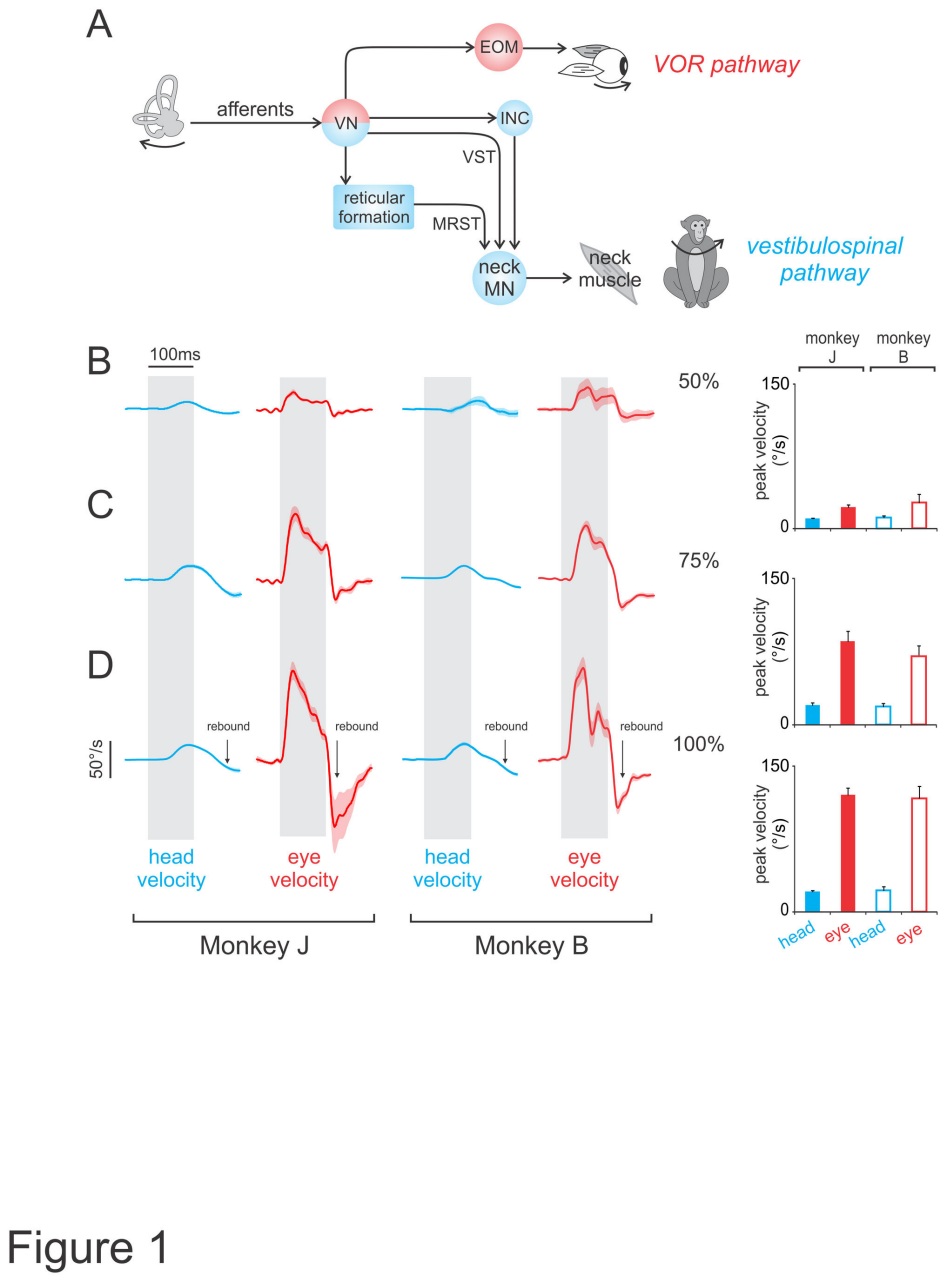
VCR and VOR responses to increasing current amplitudes. *A*, Pathways connecting the vestibular nerve to neck or extraocular motoneurons. *B*-*D*, Average head (blue) and eye (red) movement traces, in monkey J and B, evoked using current amplitudes of 50 (B), 75 (C), and 100% (D) of the maximum for pulse trains delivered at 300pps lasting 100ms. Gray bars indicate stimulus duration and shading represents standard error. Note that for some velocity traces the standard error is smaller than the line thickness. Movements away from implanted side are upwards. Arrows show rebound effect due to the release of inhibition. Insets show peak head and eye velocities for the corresponding traces. *EOM*, extraocular motoneuron; *INC*, interstitial nucleus of Cajal; *MRST*, medial reticulospinal tract; *MN*, motoneuron; *VST*, vestibulospinal tract; *VN*, vestibular nuclei.

 The vestibular system detects head motion and produces reflexive movements in response to self-motion. Specifically, the vestibulo-ocular reflex (VOR) produces compensatory eye movements to ensure stable gaze (Figure 1A, red pathway), while vestibulo-spinal reflexes ensure the maintenance of posture and balance. Interestingly, the pathways which link the vestibular periphery to neck motoneurons, should theoretically contribute to both gaze and postural stabilization (Figure 1A, blue pathway). Activation of the neck musculature produces compensatory head movements to stabilize posture (reviewed in [1]:), and since gaze motion is the sum of eye-in-head and head-in-space motion, vestibularly-driven head movements could also contribute to gaze stabilization. Indeed, in species with limited oculomotor ranges (

<±25°), robust vestibularly-driven head movements work synergistically with the VOR to extend the range of movements over which stable gaze is maintained (barn owl [2]:, cat [3]:, frog [4]:, pigeon [5,6]:, guinea pig [7]:).

To date, however, the functional significance of vestibularly-driven head movements in primates that have relatively large oculomotor ranges, larger head inertia and higher-level cognitive abilities remains a subject of controversy Head stabilization has been shown to be relatively minimal in non-human primates and humans, when subjects are passively rotated at frequencies about an earth vertical axis, consistent with a minor reflex movement response (human [8]:, squirrel monkey [9,10]:, rhesus monkey [11]:). Furthermore, modeling studies in humans suggest that the head is mainly stabilized as a result of its passive biomechanical properties [12,13]. These results are, however, in conflict with other studies showing that selective stimulation of a semicircular canal using sound [14,15] or electric current [16] can elicit substantial reflexive head rotation about the axis of that canal. Moreover, in human subjects, galvanic vestibular stimulation (GVS) consistently evokes head motion that is significantly coherent with stimulation using both single and multisine inputs [17]. Accordingly, two important questions arise: first, does vestibular prosthesis stimulation evoke head in addition to eye movements in rhesus monkeys?, and second, do these vestibularly-driven head movements contribute to gaze stabilization and head posture?

Here to address these questions, we applied short duration (100ms) stimulation to prosthetic electrodes targeted to the ampullary nerve innervating the horizontal semicircular canal [18] while simultaneously measuring evoked head movements. We systematically varied the current and rate of electrical pulse trains, and then quantified the amplitude and latency of the movements evoked during a short time period for which the contribution of longer latency voluntary inputs to the neck would be minimal. Our results 1) provide clear evidence that prosthesis stimulation can evoke head as well as eye movements in species such as rhesus monkey, and 2) suggest that evoked head movements could make a significant contribution to gaze as well as postural stability. We predict that comparable compensatory head movements will also be evoked by prosthetic stimuli in human patients. 

## Materials and Methods

### Ethics statement

All procedures were approved by The McGill University Animal Care Committee and the Johns Hopkins Animal Care and Use Committee, in addition to following the guidelines of The Canadian Council on Animal Care and the National Institutes of Health. Animals were housed in 35 ft^3^ cages and were on 12 hour light/dark cycle. Animals followed a Teklad primate diet 2055 (Harlan Inc.) based on 2-4% of body weight and received daily seasonal fruits and vegetables. Animals also had access to primate toys (Bio-Serv, Frenchtown, NJ), which were rotated on a bi-weekly basis. When animals were not being tested, they had access to play cages (67ft^3^) or enclosures (336 ft^3^) on a weekly basis. Consideration was given for the behavioral, emotional and social needs of the laboratory non-human primates when planning their housing such that compatible animals were housed in groups and provided daily enrichment as per Canadian Council on Animal Care (CCAC) guidelines. As a result, animals had opportunities to forage in a group housed setting. All surgical procedures were performed under general anesthesia using aseptic techniques. Following surgical procedures, animals were monitored and given analgesics for 2–5 days, depending on the animal's pain level.

Throughout the study, animals were monitored in consultation with the Institution's clinical veterinarian for any signs of change in their physiological and/or psychological state including: changes in responses to people, changes in levels of aggression, changes in quality and consistency of performance during experiments, changes in physical appearance (e.g. body weight and hair coat quality), loss of appetite, and the development of behavioral abnormalities (e.g. self mutilation and increased frequency of distress calls). No such changes were observed. Animals were euthanized with a method that follows the best practices recommended by the CCAC guidelines on: euthanasia of animals used in science is based on recommendations made by the International Council for Laboratory Animal Science (ICLAS) Working Group on Harmonization and the two international reference documents on euthanasia recommended by ICLAS: the American Veterinary Medical Association (AVMA) Guidelines on Euthanasia (2007) and the Working Party Report to the European Commission, Recommendations for euthanasia of experimental animals. Animals were euthanized by administering ketamine hydrochloride (15mg/kg) followed by deep pentobarbital anaesthesia (35mg/kg) and were then perfused.

### Surgical procedures

In preparation for eye and head movement recording during prosthetic stimulation, two rhesus monkeys (1 male and 1 female) underwent implantation of monocular scleral coils, a head cap, and a unilateral vestibular prosthesis. As previously described, a stainless steel post was attached to the animal's skull with stainless steel or titanium screws and dental acrylic, permitting attachment of a head coil as well as the possibility of complete immobilization of the animal's head [18,19]. Additionally, a frontal search coil was implanted to measure horizontal and vertical eye movements. To measure torsional eye movements, a second, smaller search coil (5.6 mm diameter, five turns), was implanted ~90° in relation to the frontal coil [18,20,21]. Similarly, horizontal, vertical and torsional head movements were measured using 2 orthogonally placed coils that were secured to the animal’s implant [22]. 

The vestibular prosthesis electrode arrays were implanted into the left labyrinth using a transmastoid approach [18]. First, a mastoidectomy was performed under sterile conditions. Three electrode arrays were then inserted into small openings in each semicircular canal, near the connection of the thin segment and the ampulla. The electrode arrays used in monkeys J and B were similar to those described previously in detail (see [23]). They are custom made and therefore not commercially available. The electrode array designed was based on microanatomic measurements from 3D reconstructions of CT images of existing temporal bone specimens for normal rhesus macaque monkeys. Each electrode array comprises nine active and two reference electrodes, with active electrodes partly embedded within a silicone carrier. All electrode pads and wires are 90/10 platinum/iridium alloy.

One rhesus was implanted with its canals intact (monkey J), while the other (monkey B) had received gentamicin treatment via bilateral intratympaic injection [18] to achieve reduced VOR responses consistent with bilateral profound ototoxic injury of the vestibular sensory epithelium. Intratympanic gentamicin was administered as previously described [18]. Briefly, the animal was maintained under general inhalational anesthesia (2–5% isoflurane) for 30min with the treated ear up to help ensure adequate diffusion of drug across the round window and into the inner ear. For each treatment, ^~^0.5 mL of 26.7 mg/mL buffered gentamicin solution was injected through the ear drum into the middle ear. Treatments were repeated every three weeks until VOR responses to head rotation toward the treated ear was reduced to <10% of normal gain, which required 3 injections for monkey B. Similar data were recorded from both animals, suggesting that the applied stimulation predominantly acts directly at the afferent spike initiation zone rather than via hair cells (see Discussion).

### Experimental paradigms and data acquisition

The experimental setup was designed to minimize volitional movements from influencing head motion. During recording sessions, monkeys were alert and comfortably seated in a primate chair with its heads unrestrained in complete darkness. No visual targets were presented, nor did animals perform any specific tasks during experimental sessions [10]. The level of the animal’s alertness was monitored using eye movements on-line or by watching the animal through infrared cameras. When the eye movements exhibited slow drifts and saccades were not apparent or the animal appeared drowsy through the camera, the experiment was paused and the lights were turned on in order to alert the animal. The lights were also turned on in between each stimulus condition and the animal was given food and juice reward.

The animals’ head and eye position were monitored on-line using the magnetic search coil technique as previously described [19]. Electrical stimulation was initiated only if the head and eye were stationary and centered (±10°) on the body and within the orbit, respectively. In order to avoid confounding voluntary movement, we applied stimulation for a brief (100ms) period, and focused on the responses evoked in this initial time window, preceding the time required for the generation of voluntary neck responses. Additionally, in a subset of experiments the head was restrained to allow us to compare the difference between the eye movements that were evoked with and without concurrent head movement.

Each biphasic current pulse consisted of 2 phases, each lasting 200μs. The first phase was a constant-current pulse cathodic on the “active” electrode nearest the nerve and anodic at a distant reference electrode. The second phase was a charge-balancing pulse equal and opposite that of the first phase. Maximum current amplitudes were set to 80% of the minimum value at which facial muscle activation was visible (i.e., slight blinking and contraction of facial muscles) using biphasic pulses delivered at 300 pulses per second (pps) [18]. We obtained maximum current amplitudes of 200 and 100µA for monkeys J and B, respectively. Stimulation pulse trains lasting 100ms were then delivered via the horizontal canal electrode at 50, 75 and 100% of the maximum current amplitude and pulse rates of 50, 100, 200 and 300pps. Gaze and head position were recorded using the magnetic search coil technique (CNC eye coil system with spatial resolution of ~0.01°), low-pass filtered (250 Hz, analog 8 pole Bessel filter), sampled at 1 kHz, and calibrated as described previously [18,19]. The angular position and velocity of the eye relative to the coil-frame were computed using rotation matrices, rotation vectors, and quaternions, transforming between these representations as required for computational efficiency using algebraic rotational kinematic formulae (e.g., [24,25]). A camera was used to confirm there were no facial twitch, blinks or winks during stimulation at any of the experimental current amplitudes in either animal. 

### Data analysis

Data were recorded over 4 and 8 sessions for monkeys J and B, respectively (~30 trials for each stimulation parameter). Specifically, each session was performed in one day and consisted of delivering ~20 pulse trains at each current amplitude (50, 75 100% of maximum current) and pulse rate (50, 100, 200, 300pps). We verified that maximal current amplitudes were constant across sessions, and that horizontal canal stimulation produced minimal vertical and torsional movements (<60% relative to the horizontal component). Illustrated traces of the evoked horizontal eye and head movements represent averages of data collected across sessions, with shaded bands showing standard errors across trials.

To compare eye and head movement response latencies, we first used a criterion where response onset was defined as the time at which eye or head velocity reached a value of 2 standard deviations above baseline (the average velocity 0-40ms before stimulation onset). Additionally, we used a second method based on a slope intercept criterion in which a regression line was fit to head or eye velocity over a 10ms window before the time at which peak velocity occurred. The intercept of this regression and baseline was defined as response onset. For both methods, the difference between response and stimulation onset was defined as the response latency. Values are expressed as mean ± standard error.

## Results

The goals of this study were to 1) quantify the head movements evoked by vestibular prosthesis stimulation in alert rhesus, and 2) assess whether these head movements make a significant contribution to gaze and postural stabilization. To address these goals, we stimulated prosthetic electrodes targeted to the ampullary nerve innervating the horizontal semicircular canals, and simultaneously recorded head movements and VOR eye movement responses. We then quantified these evoked eye and head responses in order to compute the contribution of each to gaze stabilization, as well as to compare their combined response with that of the VOR eye movement evoked in the head-restrained condition. 

### Quantification of vestibularly-driven head movements and VOR responses to vestibular nerve stimulation

Figure 1B-D shows average head velocity traces (blue) that were evoked using pulse trains (delivered at 300pps for 100ms) of increasing current amplitude. Consistent with a role for vestibularly-driven head motion in both head and gaze stabilization, stimulation evoked compensatory (i.e., towards the side contralateral to the stimulated nerve) head motion. Head movement amplitudes and velocities increased with increasing current amplitude. At 50% of the maximum current (Figure 1B), head movements were small (<0.25°) with velocities peaking at 9.5±1.1 and 11.2±1.8°/s, for monkeys J and B, respectively. Larger head movements were evoked at maximum current amplitudes (~1.5°) (Figure 1D), reaching velocities of 20.6±1.5 and 22.2±3.9°/s for monkeys J and B, respectively. 

Stimulation simultaneously evoked contralaterally directed eye movements (red traces in Figure 1B-D) that also increased with current amplitude (reaching ~6°). Notably, evoked eye movements were more than 5 times faster than evoked head movements, reaching peak velocities 120.7±6.8 and 117.3±12.2°/s, for monkeys J and B, respectively, at maximum current (Figure 1D). 

Note that after stimulation offset the eye movement response ceased almost instantly, and then rebounded to move in the opposite direction (see arrow in Figure 1D). In contrast, the dynamics of head movement responses were more sluggish, taking longer to stop, and also ultimately showing a less pronounced rebound effect (see arrows in Figure 1D). Notably, during the rebound portion of the eye movement the head is still moving away from the side of stimulation, in the opposite direction of the eye motion. Thus a VOR response induced by the evoked head movement could potentially contribute to the rebound eye velocity. However, given that the head movement velocity is relatively small in this interval, it is likely that the viscoelastic properties of the oculomotor plant make the major contribution to this effect. As will be shown below, this proposal is consistent with our observation that similar rebound eye movements also occurred during stimulations when the head was fixed.

**Figure 2:**
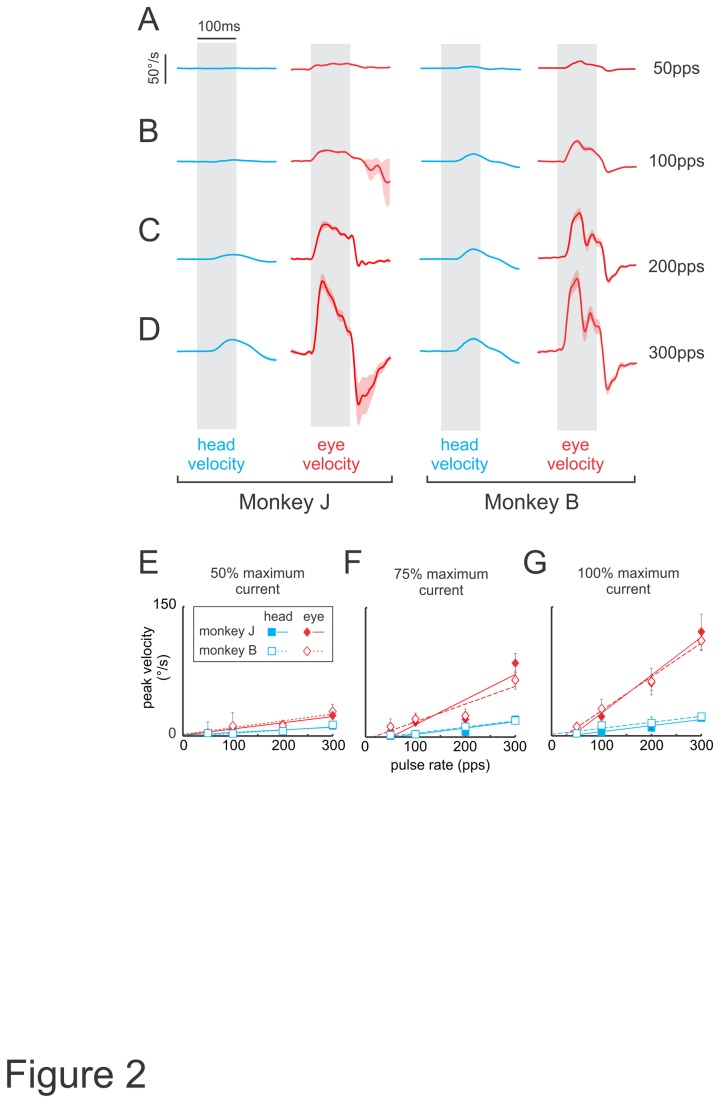
Average VCR and VOR responses to increasing pulse rates. *A*-*D*, Average head (blue) and eye (red) movement traces, in monkey J and B, evoked using increasing pulse rates of 50 (A), 100 (B), 200 (C) and 300pps (D) at maximum current amplitude. *E*-*G*, Plots of peak head and eye velocities as a function of pulse rate for current amplitudes of 50 (E), 75 (F), 100% (G) of the maximum.

To further characterize the relationship between evoked eye and head movements, we next varied pulse rate as a parameter. Movement amplitudes and velocities increased with pulse rate when current amplitude (100% of maximum current) and stimulation duration (100ms) were held constant. Figures 2A-D show the average head (blue) and eye (red) velocity traces evoked for pulse rates (50-300pps), which were chosen to effectively span the physiological range of vestibular afferent firing rates [26]. Figure 2E-G summarizes the relationship between the stimulus pulse rate and the peak movement velocity elicited at 3 different current amplitudes. As illustrated in Figure 2A-D, peak head velocity increased when the pulse rate was increased from 50-300pps, regardless of the current amplitude used (see blue squares in Figure 2E-G). Similarly, for a fixed pulse rate, increases in current amplitude also evoked increasingly faster head movements. Correspondingly, peak eye velocity increased as a function of both pulse rate (red diamonds in Figure 2E-G) and current amplitude (e.g., compare red diamonds for stimulations delivered at 300pps in Figure 2E-G). Notably, the peak velocities of evoked head movements were smaller than peak eye velocities (compare blue squared and red diamonds in Figure 2E-G) and in all conditions, peak eye and head velocities remained the same across different sessions (P>0.05; Figures 1 and 2). Taken together, our analysis revealed that head velocities were reliably evoked but were consistently smaller than eye velocities across all tested stimulus conditions.

Although, the eye and head movements were mostly in the plane of the horizontal canal, vertical and torsional movement components were also observed (<60% relative to the horizontal component). The vertical and torsional components are most likely due to current spread to afferents innervating the posterior or anterior canals. Misalignment of VOR responses during vestibular prosthesis stimulation has been previously reported in rhesus monkey and chinchilla. Notably, as detailed in Dai et al. [18], chinchillas exhibit significantly greater misalignment than rhesus monkey, possibly due to enhanced ampullary nerve stimulation selectivity in rhesus monkey since their labyrinths are approximately 1.5-1.7 times larger than those of the chinchilla and allow for relatively more precise electrode placement and greater separation between afferent nerve branches. If misalignment is inversely related to labyrinth dimensions as results in chinchillas and rhesus monkeys suggest, then it should be further reduced when the device is transitioned to humans, because the dimensions of the human labyrinth are significantly larger than both chinchillas and rhesus monkeys [27–30]. 

### Quantification of the latency of evoked vestibularly-driven head movements versus VOR responses and their relative contributions to gaze stabilization

**Figure 3:**
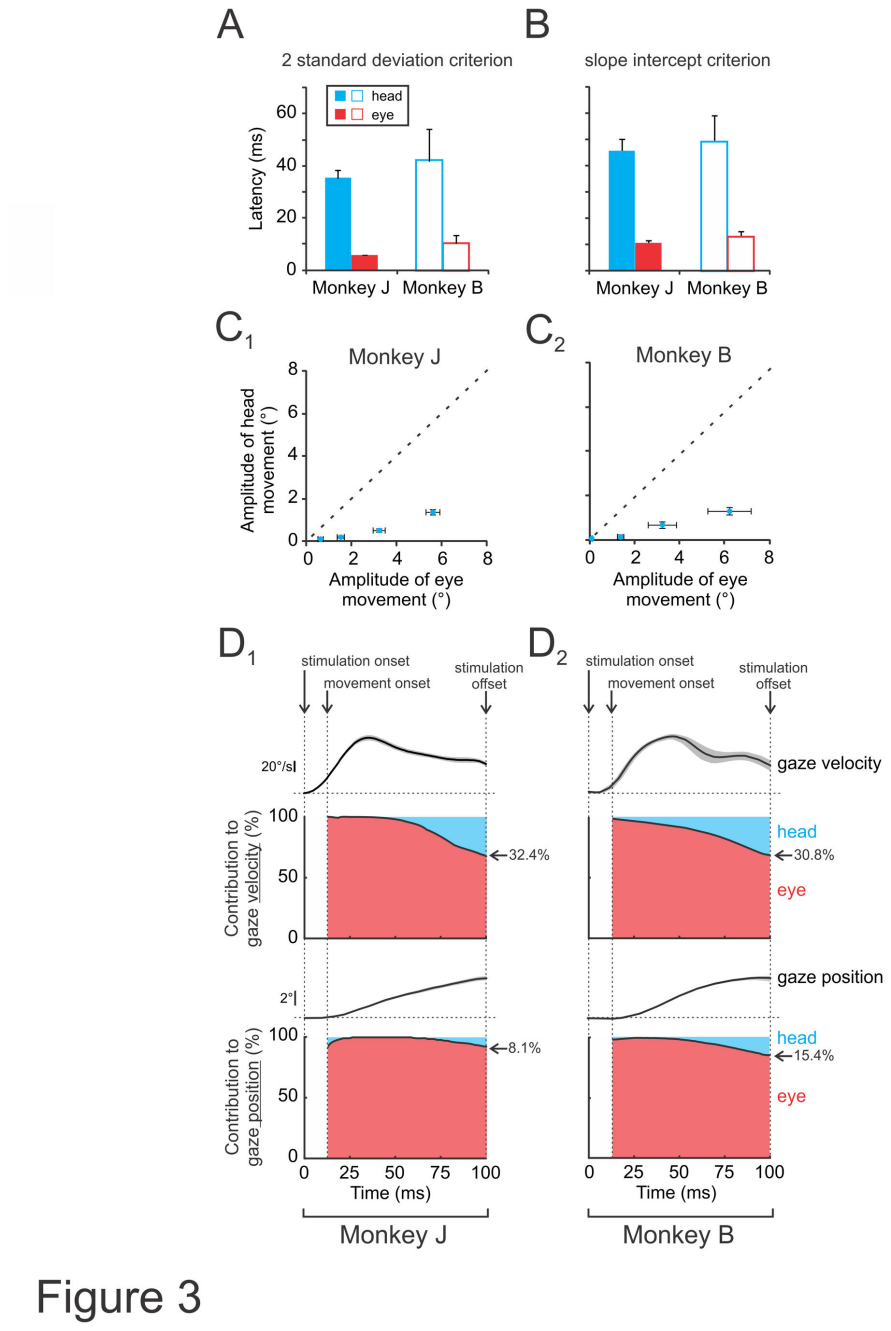
VCR and VOR response latency and relative contribution to gaze. *A*-*B*, Latency of evoked eye or head movements using a 2 standard deviation (A) or slope intercept measurement (B). *C*, Plots of eye versus head movement amplitude during pulse trains delivered at 50, 100, 200 and 300pps. *D*, Top panels show the contribution of head and eye to instantaneous gaze velocity during pulse trains delivered at the maximum current amplitude and 300pps. Bottom traces show the contribution of head and eye to cumulative gaze position. The average gaze velocity (top panels) and position (bottoms panels) traces are also plotted for comparison.

During self-motion, the relative motion of the world would induce blurred vision without the presence of vestibular pathways that rapidly stabilize gaze. The VOR, which has a fast response time (5-6ms [31]), has long been known to play a vital role in maintaining stable gaze during everyday activities. To further characterize the vestibularly-driven head movements evoked by vestibular nerve stimulation we next quantified their response latency. In order to ensure the best signal-to-noise ratio (i.e., largest head velocity response), this analysis was performed on the responses evoked by stimulation at maximum current amplitude (see Materials and Methods) and pulse rate (300pps). Response latencies were first determined using a 2 standard deviation criterion (see Materials and Methods). On average, head movements were only apparent well after the stimulus onset (35.2±3.1 and 41.8±12.3ms for monkey J and B, respectively; Figure 3A), in contrast to eye movements, which were initiated almost immediately following stimulus onset (5.8±0.2 and 10.3±3.1ms for monkey J and B, respectively; Figure 3A). Comparable results were obtained using a second criterion (slope intercept criterion, see Materials and Methods; Figure 3B) confirming the robustness of the latency estimates, and eye and head movement latencies remained the same across different sessions (P>0.05). Interestingly, the latency of evoked head movements while longer than VOR eye movements, was shorter than those of visual pursuit pathways in rhesus monkeys [32,33], and thus head motion could potentially contribute to gaze as well as postural stabilization.

Next to determine whether these same evoked head movements made a significant contribution to stabilizing gaze, we quantified their contribution relative to that of the VOR. Figures 3C_1_ and 3C_2_ plot head versus eye displacement produced by stimulation in both monkeys. All data points fall well below the unity line, indicating that evoked head movement amplitudes were much smaller then evoked eye movement amplitudes. Figures 3D_1_ and 3D_2_ plot the time varying head and eye contribution to instantaneous gaze velocity (top panels) and to cumulative gaze position (bottom panels). The average gaze velocity (top panels) and position (bottoms panels) traces are also plotted for comparison. Note that, vestibularly-driven head movements initially made little contribution to the overall gaze motion as compared to eye movements (VOR) due to both longer latencies and smaller amplitudes. However, by ~100ms after stimulation onset, head movements contributed >30% of gaze velocity. Thus, in comparison to the VOR, which produces rapid compensatory eye movements within 5-6ms, vestibularly-evoked head movements made an increasingly significant contribution to gaze stabilization over a longer time scale.

**Figure 4:**
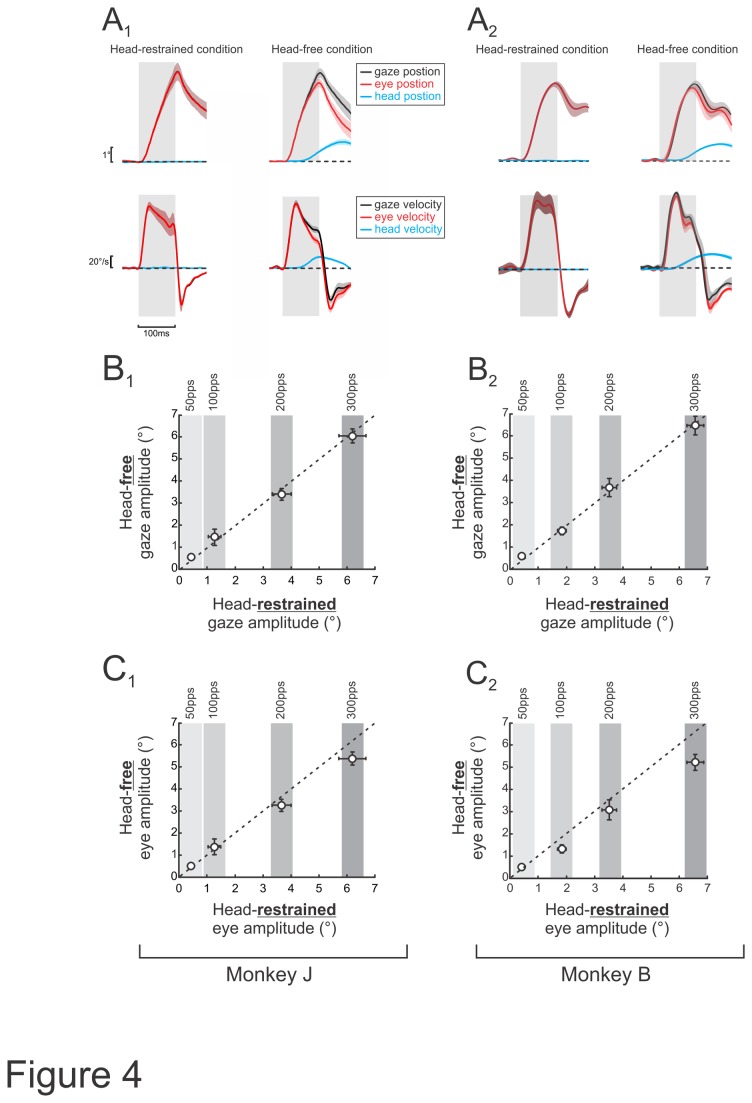
Interaction of vestibular-driven head and eye movements. *A*, Average gaze, eye and head position (top panels) and velocity (bottom panels) traces during stimulations when the head was restrained and free. Gray bars indicate stimulus duration and shading represents standard error. *B*, Plots of average gaze movement amplitude during pulse trains delivered at 50, 100, 200 and 300pps when head-restrained versus free. *D*, Plots of average eye movement amplitude during pulse trains delivered at 50, 100, 200 and 300pps when head-restrained versus free.

Finally, in order to determine how the vestibularly-driven head movement and VOR eye movements interact, we completed a series of stimulations when the head was restrained (using identical parameters as above). We then compared the eye movements evoked when the head was restrained to those evoked by comparable stimulation applied when the head was free to move. Figure 4A illustrated the average eye, head and gaze position and velocity traces during these 2 conditions (maximum current amplitude and 300pps). Notably, total gaze amplitude was equivalent in both conditions, and thus was unaffected by the head’s ability to move (Figure 4B). Thus, correspondingly, when the head was free to move, the evoked eye movement amplitude was smaller than in the condition where the head was restrained (Figure 4C). Taken together, these results suggest that vestibularly-evoked head movements and the VOR work together to stabilize gaze in space. Specifically, when the head is restrained, the applied electrical stimulation causes VOR eye movements towards the side contralateral to the stimulated nerve. In contrast, when the head is allowed to move freely, the applied stimulation induces head motion towards to the side contralateral to the stimulated nerve. This head movement secondarily causes a small VOR response component in the opposite direction (i.e., towards the side of the stimulated nerve), effectively decreasing the net amplitude by which the eyes move. Therefore, the eye-in-head movement amplitude when the head is free to move is less because of the interaction with the secondary VOR induced by the evoked head movement. Finally, note that similar results were observed in both animals suggesting that sensory substitution (i.e. neck proprioceptive stimulation produced by vestibularly-evoked head movements [34]) may have helped compensate for the deficient vestibular input in the animal that received bilateral gentamicin treatment (monkey B). Since monkey J has an intact vestibular system and monkey B received bilateral gentamicin treatment, the secondary VOR was generated by different mechanisms in each animal. In monkey J, the evoked head movement can be expected to elicit a VOR response in the direction opposite of the head movement and therefore opposite to the direction of the initial eye movement response to the applied stimulation. This would result in a net decrease in the eye movement. In both monkeys, the head movement activated neck proprioceptors since the head rotated relative to the body. This sensory input has been shown to substitute for vestibular input in animals that have been rendered bilaterally vestibular deficient [34]. Thus, the secondary “VOR-like” response recorded in monkey B was most likely due to the activation of neck proprioceptors. We note that this difference in the secondary VOR response may explain the difference in trajectory of the evoked eye movements (Figures 1B-D and 2A-D): while monkey J exhibits a monophasic deflection in the eye velocity, monkey B’s eye movements are biphasic.

## Discussion

In the present study, we quantified the head movements evoked in alert rhesus monkey by vestibular prosthesis stimulation. We applied short pulse trains (100ms) and characterized the head movements evoked during this initial time period for which the contribution of longer latency voluntary inputs to the neck would be minimal. We first found that the head movements evoked by prosthesis stimulation were characterized by longer latencies and smaller amplitudes compared to simultaneously evoked VOR eye movement responses. We then quantified the relative contribution of these evoked head movements to gaze stabilization, and found that in comparison to the VOR, the head movements evoked by our stimulation significantly contributed to gaze stabilization after sustained stimulation (>30% of gaze velocity, after 100ms). Finally, to understand the interaction between evoked head and eye movements, we applied the same stimulation in the head-restrained condition and measured the VOR response. We found no difference in the total gaze response (i.e., head-in-space + eye-in-orbit) evoked during the head-restrained and head-free conditions, demonstrating that the sum of evoked eye and head movements remained constant. Thus, taken together our results provide evidence that prosthesis stimulation can evoke vestibularly-driven head motion, which contributes to gaze and postural stabilization. We speculate that analogous compensatory head movements will likely be evoked in human patients implanted with vestibular prostheses.

### Comparisons with previous studies

Here we have shown that the compensatory head movement response evoked by prosthetic electrical stimulation is small but significant in rhesus monkeys. This result is consistent with a recent report, showing that GVS evokes significant head motion in normal human subjects [17]. In contrast, prior studies using applied rotational stimulation have reported negligible compensatory head movement responses in rhesus monkeys [11], and humans [8]. There are at least two possible explanations for the apparent discrepancy. First, the stimuli used in the present study provided a robust vestibular input that included a transient onset, while the rotational stimuli applied in these previous studies were steady state sinusoidal rotations (typically applied by passively rotating the whole body). Modeling studies, however, have suggested that the vestibularly-driven head movements would be most beneficial at relatively low frequencies (i.e., <2Hz) due to the head’s inertial properties [10,12,13,35]. A second and more likely possibility is that humans and rhesus monkeys actively suppress compensatory head motion particularly during passively applied low frequency rotations [12,13]. Importantly the ability of monkeys to actively supress vestibularly-driven head movements is avoided using the unexpected transient stimulation we applied here.

Interestingly, recent evidence from studies in rhesus monkeys during locomotion suggests that vestibular-neck pathways can be important for stabilizing the head during actively-generated movements in primates [36,37]. Further experiments combining behaviour and neurophysiological approaches during natural voluntary behaviours will be required to dissociate how voluntary self-motion and vestibularly-driven head motion interact in everyday life. 

### Neural correlates of evoked eye and head movement responses

Eye movement latencies measured in this study are consistent with the known mechanical and signal transduction delays of the direct VOR pathway connecting vestibular afferents to extraocular motoneurons (Figure 1A, red). Comparable latencies have been measured in response to high acceleration transients [31]. The relatively longer latencies of evoked head motion are similarly consistent with previous studies of the pathways connecting the vestibular labyrinth to the cervical spinal cord to mediate the vestibulo-collic reflex (VCR). Notably, vestibular evoked myogenic potentials (VEMPs) first appear 13ms following brief clicks played through headphones (i.e., p13-n23 [38]). These responses are abolished following vestibular neurectomy, confirming they are driven by vestibular reflexes (see Figure 7 in [38]). Thus, if one accounts for the time required for the head to actually move following neck motoneuron activation (>20ms [39]) as a result of the relatively sluggish dynamics of the neck versus eye plant [12,13,40], the latencies we measure in response to prosthetic stimulation are consistent with existing data. 

Electrophysiological evidence further suggests that the dominant pathway(s) mediating the VCR include indirect pathways involving additional relay structures such as the interstitial nucleus of Cajal and reticular formation as well as direct projections from the vestibular nuclei to the neck motoneurons (Figure 1A, blue; reviewed in [41]:). Because the relative difference in the latencies of these pathways would only be on the order of a few milliseconds, it was not possible to definitely parse their relative contributions in the present study since the latencies of head movements (rather than motoneuron activation) were measured. Nevertheless, the latencies inherent to these pathways would also be consistent with those reported here. 

It is important to consider that the head-on-body rotations evoked by our stimulation would have activated proprioceptors within the neck musculature. Thus the resultant activation could, in turn, induce the cervico-collic reflex (CCR); a reflex that would theoretically oppose head rotations produced by the VCR, to potentially stabilize the inertia of the head relative to the body (cat [42]:, squirrel monkey [10,43]:). Studies in humans, however, suggest that VCR is an order of magnitude stronger than the CCR in human [8,13]. Nevertheless, it has been proposed that the CCR could be facilitated after labyrinthine loss [44] and thus could have potentially functioned to reduce the head movements evoked by vestibular stimulation in our animals.

It is also noteworthy that head movements evoked by our targeted stimulation of the horizontal ampullary nerve were consistent with vestibular activation rather than the acoustic startle reflex. The latter reflex is driven by a sudden intense auditory stimulus and serves to protect the head and body from impact stimuli. These evoked head movements are characterized by longer latencies (>80 ms) and neck EMG generating ipsilateral rather than contralateral head movement [45]. Furthermore, consistent with our findings, sound-induced vestibular stimulation in human patients evokes head movements in the plane of the fenestrated canal [15]. We predict that future experiments using stimulating electrodes in anterior or posterior semicircular canals should produce head movements in the plane of each targeted canal. 

### Electrical Stimulation: Functional Considerations and Mechanisms of Activation

One benefit of the technique used in the present study, is that we were able to apply vestibular stimulation without actually moving the head. Accordingly, our approach allowed the dissociation between input (i.e., semicircular canal activation) and output (neck motion) in a manner that is not possible using natural stimuli where extracting the vestibularly evoked motion depends on the biomechanical properties of the head/neck plant. Regardless of whether motion is transiently (e.g., [46]) or continually [8,11] applied. Here, electrical stimulation was applied via metal electrodes implanted within the labyrinth using an approach similar to that of recent studies by several groups [18,47,48]. This type of stimulation is thought to predominantly act at the spike initiator zone of primary vestibular afferents, although modulation of hair cell transmembrane potential and consequent changes in neurotransmitter release may also contribute [49,50]. Indeed, our observation that similar data were recorded from both animals even though one was implanted with its canals intact, while the other had received gentamycin treatment (see Methods), provides further support for the proposal that stimulation predominantly acts directly at the spike initiator zone. Fibers characterized by more irregular spontaneous discharge and/or larger axonal caliber are preferentially recruited at lower stimulus currents, but a wider population of fibers is likely activated by stimuli well above the threshold at which both VOR and head movement responses becomes apparent [49,51]. 

### Implications for future work

It is generally believed that the efficacy of head motion responses driven by vestibular stimulation is reduced in higher mammals which require precise voluntary control. Our results, however, show that significant vestibular-driven compensatory head movement can be evoked in the rhesus monkey. In species such as humans and primates, head movements are often made purposefully, for instance to redirect gaze. A scenario in which activation of the neck musculature is dominated by a vestibularly-driven reflex would not be behaviourally advantageous in this context, since these compensatory movements would hinder intended voluntary movement. Indeed, while vestibulo-spinal interneurons are robustly modulated during passive motion in rhesus monkeys, they are selectively attenuated during active movements [52,53]. The reduction in neural sensitivity is consistent with the above proposal, since a vestibularly-driven commanded head movement would actually be counterproductive when the goal is to produce voluntary head movement. Further work will be required to determine the efficacy of neuronal pathways mediating compensatory head movement during natural activities such as gaze shifts and locomotion. 
